# Glomerulopathies in Brazil: 25 years of epidemiological change and emerging challenges

**DOI:** 10.1590/2175-8239-JBN-2025-E017en

**Published:** 2025-12-08

**Authors:** Stanley de Almeida Araújo, David Campos Wanderley

**Affiliations:** 1 Instituto de Nefropatologia LTDA-ME, Belo Horizonte, MG, Brazil.; 2 Universidade Federal de Minais Gerais, Centro de Microscopia Eletrônica, Belo Horizonte, MG, Brazil.; 3 Faculdade de Medicina Ciências Médicas de Minas Gerais, Departamento de Anatomia Patológica, Belo Horizonte, MG, Brazil.

Glomerulopathies constitute a substantial proportion of chronic kidney disease (CKD) worldwide and, in Brazil, represent a critical intersection between science, public health, and social equity. In a country of continental dimensions, marked by profound regional inequalities and a heterogeneous epidemiological profile, long-term, reliable data series are essential to guide clinical practice and health policy decisions. In this context, the study by Sales et al.^1^, encompassing 25 years of follow-up at a major university referral center in Minas Gerais, is more than a scientific contribution—it serves as a diagnostic lens into contemporary Brazilian nephrology.

The analyzed cohort included 417 adult patients followed over 25 years (1998–2023), with a mean age of 41.7 years and a predominance of women (57.3%). Among primary glomerulopathies, membranous nephropathy emerged as the most prevalent subtype, while lupus nephritis remained the leading secondary diagnosis. This distribution contrasts with classic Brazilian surveys, which historically identified focal segmental glomerulosclerosis (FSGS) as the most frequent primary glomerulopathy^2,3,4^. Such divergence may reflect true changes in disease spectrum—driven by demographic shifts (population aging and improved survival of patients with autoimmune diseases), environmental influences, and therapeutic advances. However, it may also be related to evolving policies and practices for kidney biopsy indication, broader access to complementary tests, and enhanced local diagnostic capacity. A noteworthy counterpoint comes from our experience at the *Instituto de Nefropatologia* (Belo Horizonte, Minas Gerais), which has evaluated approximately 33,000 kidney biopsies over 15 years. In this large series, IgA nephropathy (IgAN) remains the most prevalent primary glomerulopathy^5^, reflecting both the regional epidemiologic profile and the availability of appropriately indicated biopsies for the investigation of persistent hematuria and proteinuria. This finding underscores how organizational factors and access to diagnostic procedures shape the epidemiological landscape of glomerulopathies, emphasizing the importance of interpreting historical series within their local context and the structure of referral services.

From a prognostic standpoint, the results provide clear and clinically actionable insights: minimal change disease was associated with the best renal survival, whereas membranoproliferative glomerulonephritis correlated with poorer outcomes. Importantly, the study identified both the presence of tubulointerstitial fibrosis/atrophy (IFTA) and elevated body mass index (BMI) as independent predictors of CKD progression. The emergence of obesity—and BMI as a prognostic variable—confirms that modern nephrology must integrate with the broader metabolic health agenda and preventive strategies, recognizing that modifiable systemic factors decisively influence renal outcomes^6^.

Temporal trend analysis revealed noteworthy patterns: an increase in lupus nephritis, a decline in podocytopathies, and relative stability of IgA nephropathy. These trends partially align with observations from the International Kidney Biopsy Survey^7^ and Latin American registries^8^ but acquire unique nuances when viewed regionally: disparities in biopsy access, diagnostic delays, and social determinants of health shape disease prevalence and clinical presentation across Brazil. Consequently, generalized recommendations require local adaptation, and screening strategies must prioritize high-risk regions and populations.

From a practical perspective, the study reinforces three priority actions. First, the early indication of kidney biopsy in patients with persistent hematuria and proteinuria—even if subnephrotic—to prevent irreversible chronic injury. Second, the integration of renal care with weight management, glycemic control, and cardiovascular prevention programs^9^. Third, the development of collaborative care protocols between nephrologists and rheumatologists, ensuring timely access to modern immunomodulatory therapies, including biologic agents when indicated^10^.

The study’s retrospective design entails limitations, including loss to follow-up, heterogeneity of clinical documentation, and evolving diagnostic and therapeutic criteria over 25 years. Nonetheless, these constraints do not diminish the value of the series; rather, they highlight the urgent need for prospective, multicenter studies with biobanks and standardized collection of clinical, histological, and molecular data.

Additionally, the article reveals an inescapable dimension: the impact of socioeconomic inequalities and health system organization on the natural history of glomerulopathies. In regions where access to primary and secondary/tertiary care is limited, patients often present late, when kidney damage and loss of function are already irreversible, leaving renal replacement therapy as the only viable option. Interventions successfully implemented in other countries—such as systematic screening for proteinuria in at-risk populations, integrated obesity and diabetes management programs, and streamlined clinical pathways linking primary care to specialized centers^11^—can and should be adapted to Brazil’s Unified Health System (SUS).

We propose pragmatic goals: reduce by 20% biopsies performed at advanced CKD stages within the next five years; implement regional BMI control programs for patients diagnosed with glomerulopathies; and establish a national consortium to standardize histopathology reporting and data collection. The effective implementation of these strategies requires the recognition and formal incorporation of complementary diagnostic methods—including special stains, immunofluorescence, immunohistochemistry, and electron microscopy—resources that, surprisingly, remain officially absent from Brazil’s SUS. Although some centers can perform these analyses, there is currently no specific registration or coding for these procedures in the SIGTAP system (*Sistema de Gerenciamento da Tabela de Procedimentos, Medicamentos e OPM do SUS*). This prevents appropriate reimbursements, hinders resource allocation, and makes it impossible to generate robust epidemiological data. In parallel, sustained investments are needed in the training and continuing education of pathologists and nephrologists, in the strengthening of laboratory infrastructure, and in the funding of translational research that integrates clinical, histopathological, and molecular data to advance the diagnosis, prognosis, and management of glomerulopathies.

The study by Sales et al. plays a dual role: it chronicles 25 years of transformation and ignites an agenda for change. Understanding the past is not enough; knowledge must be translated into action— expanding access to diagnostic biopsy, fostering multidisciplinary networks, integrating metabolic risk management into renal care, and investing in molecular diagnostics^6,12^. This is the path towards reducing the burden of glomerulopathies in the 21st century and ensuring that every data point translates into improved outcomes and greater equity among Brazilian patients.

## Data Availability

The raw data of the study can be accessed by researchers from the sources provided.
