# Supplementary material for: Glomerulopathies in Brazil: 25 years of epidemiological change and emerging challenges
Source: J Bras Nefrol. 2025 Dec 8;47(4):e2025E017. doi: 10.1590/2175-8239-JBN-2025-E017en (PMC12698066; doi:10.1590/2175-8239-JBN-2025-E017en)
Supplement: Translation

# Translation: Glomerulopatias no Brasil: 25 anos de mudanças epidemiológicas e novos desafios

**DOI:** 10.1590/2175-8239-JBN-2025-E017pt

**Authors:** Stanley de Almeida Araújo, David Campos Wanderley

**Affiliations:** 1 Instituto de Nefropatologia LTDA-ME, Belo Horizonte, MG, Brasil.; 2 Universidade Federal de Minais Gerais, Centro de Microscopia Eletrônica, Belo Horizonte, MG, Brasil.; 3 Faculdade de Medicina Ciências Médicas de Minas Gerais, Departamento de Anatomia Patológica, Belo Horizonte, MG, Brasil.

As glomerulopatias constituem uma parcela significativa das doenças renais crônicas (DRC) em todo o mundo e, no Brasil, representam um ponto de encontro entre ciência, saúde pública e equidade social. Em um país de dimensões continentais, marcado por desigualdades regionais profundas e por um padrão epidemiológico heterogêneo, o Brasil necessita de séries históricas longas e confiáveis para orientar práticas clínicas e decisões de políticas. Nesse cenário, o estudo de Sales et al.^1^, com 25 anos de seguimento em um centro universitário de referência em Minas Gerais, constitui não apenas uma contribuição científica, mas também um instrumento de diagnóstico da nefrologia brasileira contemporânea.

A coorte analisada abrangeu 417 pacientes adultos acompanhados ao longo de 25 anos (1998–2023), com idade média de 41,7 anos e predomínio do sexo feminino (57,3%). Entre as glomerulopatias primárias, a nefropatia membranosa destacou-se como a entidade mais prevalente, enquanto, entre as formas secundárias, a nefrite lúpica se manteve como o principal diagnóstico. Essa distribuição contrasta com levantamentos brasileiros clássicos, que historicamente apontavam a glomeruloesclerose segmentar e focal (GESF) como a glomerulopatia primária mais frequente^2,3,4^. Essa diferença pode refletir transformações reais no espectro de doenças, impulsionadas por mudanças demográficas (como envelhecimento populacional e aumento da sobrevida de pacientes com doenças autoimunes), fatores ambientais e avanços terapêuticos. No entanto, também é plausível que esteja relacionada a alterações nas políticas e práticas de indicação de biópsia renal, à ampliação do acesso a exames complementares e à evolução da capacidade diagnóstica local. Um contraponto marcante vem da nossa experiência no Instituto de Nefropatologia (BH/MG), que, em 15 anos, avaliou aproximadamente 33 mil biópsias renais, nas quais a nefropatia por IgA (NIgA) permanece como a glomerulopatia primária mais prevalente^5^, refletindo tanto o perfil epidemiológico regional quanto o acesso à biópsia (adequadamente indicada) para investigação de hematúria e proteinúria persistentes. Esse dado reforça como aspectos organizacionais e de acesso ao diagnóstico moldam o retrato epidemiológico das glomerulopatias, sendo fundamental interpretar séries históricas à luz do contexto local e da estrutura dos serviços de referência.

No plano prognóstico, os resultados oferecem lições claras e aplicáveis tanto ao consultório quanto à gestão: a doença de lesões mínimas apresentou melhor sobrevida renal, enquanto a glomerulonefrite membranoproliferativa esteve associada a piores desfechos. De modo crucial, o estudo identificou a presença de fibrose túbulo-intersticial/atrofia tubular (IFTA) e o índice de massa corporal (IMC) elevado como preditores independentes de progressão para DRC. A emergência da obesidade e, consequentemente, do IMC como variável prognóstica, confirma que a nefrologia moderna deve se articular com a agenda metabólica e com políticas de prevenção desse problema atual, reconhecendo que fatores sistêmicos modificáveis influenciam decisivamente a evolução renal^6^.

A análise temporal revelou padrões instigantes: aumento da nefrite lúpica, declínio das podocitopatias e relativa estabilidade da nefropatia por IgA. Esses movimentos convergem parcialmente com observações do *International Kidney Biopsy Survey* ^7^ e de registros latino-americanos^8^, mas ganham contornos próprios quando vistos sob a ótica regional: a variabilidade no acesso à biópsia, o atraso no diagnóstico e os determinantes sociais da saúde moldam a prevalência e a apresentação clínica em diferentes territórios brasileiros. Consequentemente, recomendações gerais devem ser adaptadas localmente, e as estratégias de rastreamento devem priorizar regiões e populações de maior risco.

Do ponto de vista prático, o trabalho reforça três ações prioritárias. Primeiro, a indicação precoce de biópsia renal em pacientes com hematúria persistente e proteinúria—ainda que subnefrótica—, a fim de evitar que o dano se perpetue, tornandose crônico e irreversível. Segundo, a integração do cuidado renal com programas de gestão do peso, controle glicêmico e prevenção cardiovascular^9^. Terceiro, protocolos de colaboração entre nefrologistas e reumatologistas, com acesso célere a terapias imunomoduladoras modernas, incluindo agentes biológicos quando indicados^10^.

As limitações do estudo, inerentes ao desenho retrospectivo, incluem perda de seguimento, heterogeneidade da documentação clínica e mudanças nos critérios diagnósticos e terapêuticos ao longo de 25 anos. Todavia, essas restrições não diminuem o valor da série; ao contrário, sublinham a urgência de estudos prospectivos multicêntricos, com biobancos e coleta padronizada de dados clínicos, histológicos e moleculares.

Além disso, há uma dimensão inadiável que o artigo ajuda a revelar: o efeito das desigualdades socioeconômicas e da organização do sistema de saúde sobre a história natural das glomerulopatias. Nas áreas em que o acesso à atenção básica e aos níveis secundários/terciários de cuidado é restrito, o paciente frequentemente chega tardiamente, quando o processo de adoecimento e a perda das funções renais já são irreversíveis, restando apenas a indicação de Terapia Renal Substitutiva. Intervenções que funcionaram em outros países, como a triagem sistemática de proteinúria em populações de risco, programas integrados de manejo da obesidade e do diabetes, bem como caminhos clínicos rápidos entre a atenção primária e os centros especializados^11^, podem e devem ser adaptadas à realidade do SUS. Propomos metas pragmáticas: reduzir em 20% a proporção de biópsias realizadas em estágios avançados nos próximos cinco anos, implementar programas regionais de controle do IMC em pacientes com diagnóstico de glomerulopatia e criar um consórcio nacional para padronização de laudos histopatológicos e de coleta de dados.

A efetiva implementação dessas estratégias exige o reconhecimento e a incorporação adequada de métodos diagnósticos complementares (colorações especiais, imunofluorescência, imuno-histoquímica e microscopia eletrônica), recursos que, surpreendentemente, ainda não estão formalmente contemplados no Sistema Único de Saúde (SUS). Embora alguns serviços consigam realizar tais análises, não há, atualmente, registro ou codificação específica para esses procedimentos no SIGTAP (Sistema de Gerenciamento da Tabela de Procedimentos, Medicamentos e OPM do SUS), o que impede seu faturamento adequado, dificulta a alocação de recursos e inviabiliza a geração de dados epidemiológicos consistentes. Além disso, são indispensáveis investimentos contínuos na formação e atualização de patologistas e nefrologistas, no fortalecimento da infraestrutura laboratorial e no financiamento de pesquisas translacionais que integrem informações clínicas, histopatológicas e moleculares, favorecendo avanços no diagnóstico, no prognóstico e no manejo das glomerulopatias.

O estudo de Sales et al. cumpre papel duplo: registra uma trajetória de 25 anos e aciona uma agenda de transformação. Não basta conhecer o passado; é necessário traduzir conhecimento em ação—expandir o acesso à biópsia diagnóstica, desenvolver redes multiprofissionais, integrar a gestão metabólica ao cuidado renal e investir em diagnósticos moleculares^6,12^. Essa é a estrada para reduzir a carga das glomerulopatias no século XXI e garantir que cada dado coletado se converta em melhor prognóstico e mais equidade para os pacientes brasileiros.

## Disponibilidade de Dados

Os dados brutos do estudo podem ser acessados pelos pesquisadores nas fontes informadas.
